# Heroism in Hospital Workers

**Published:** 1915-10-30

**Authors:** 


The Workers' Newspaper of Administrative Medicine and Institutional Life, Administration, National Insurance and Health.

No. 1533, Vol. LIX. SATURDAY, OCTOBER 30, 1915. PRICE ONE PENNY.

### HEROISM IN HOSPITAL WORKERS.

In our issue of February 27 last we published an article on "Military Medical Heroism." In that article it was pointed out that honours for active and open valour are less within the ambition of the Medical Service than of any other branch of the Army. The need for preventing recklessness among the medical men has been impressed upon the nation by the losses entailed on them in the earlier days of the campaign. Medical service in the field enforces an apprehension of the meaning of true responsibility and self-control. Medical men on service in war-time earn fame, indeed, as much by the risks which they have avoided as by those they have equally bravely undergone. Yet all the time medical officers at the Front have to face terrible facts, and often indescribable horrors combined with unlimited risks, though they are free from any participation in the active fighting which is the soldier's work and duty.

Medical heroism is not, however, confined to experience in war. The war against disease, especially in the time of epidemics, calls frequently for the exhibition of continuous courage arising from a knowledgeable risk of life, daily and hourly faced by the medical profession. Those members of the medical profession who voluntarily went to Serbia to fight typhus and variola of the most malignant types, who had to handle and treat patients under the most insanitary, unwholesome, and disgusting surroundings, who were called upon to continue for the greater part of each twenty-four hours in work for the sick in these awe-inspiring and unnerving conditions, and who, despite all the set-backs and difficulties, with appalling mortality amongst the patients, did their duty without flinching and gave of themselves to the utmost. Whilst devoting themselves entirely and continuously to the discharge of their onerous fight with disease and death, they exhibited a bravery and devotion which could not be surpassed by any human being on the most strenuous of battlefields. Within our experience in the past, when hygiene was in its infancy, and the means of handling epidemic disease in large populations were few and imperfect, the mortality amongst members of the profession in the worst and greatest epidemics proved sometimes so serious as to reduce one by one the number of medical workers almost to the vanishing point. In such a case the call for courage and devotion on the part of the medical workers necessarily proves as strenuous and physically depressing as any situation which man can be called upon to face. Military medical heroism may be rewarded by the possession of the Victoria Cross, but medical heroism pure and simple during epidemics amongst the civil population has never received any similar honours or recognition.

We are led to recall these types of heroism amongst doctors by the cruel murder by the ruthless Germans of the martyr-nurse Edith Louisa Cavell. In the present day, except on the battlefield, medical heroism is necessarily accompanied by the co-operation and courageous work of devoted women, many of whom have given up all worldly ambitions in order to place their services as nurses at the disposal of the wounded. In epidemics—and this was notably so in the Serbian instances just mentioned—many brave women volunteered to accompany the doctors, and to share the risks, dangers, and discomforts of labouring with them, throughout the appalling epidemics there, to rescue the lives of as many innocent victims as possible. It is not unnatural that there should be a growing feeling amongst the public, and an ever-increasing desire on the part of those in a position best to appreciate it, that public recognition should be made of the devotion and heroism of women workers in the base hospitals in war-time, in the disease centres during epidemics, and in isolated instances where the life or death of a patient rests mainly in the hands of the trained nurse.

The *Nursing Mirror* has voiced this feeling by suggesting the creation of a Cavell Cross for heroism by women of our race and Empire. We hope this suggestion may find favour with His Majesty the King, and that it may lead to the creation of such an Order, which would, in fact, be the most fitting memorial of the martyr-nurse whose heroism and death have won for her immortal fame.

